# NLRP3 inflammasome signaling as an early molecular response is negatively controlled by miR-186 in CFA-induced prosopalgia mice

**DOI:** 10.1590/1414-431X20187602

**Published:** 2018-07-16

**Authors:** Ming-lei Chen, Kang Lin, Shu-kai Lin

**Affiliations:** 1 Department of Neurology, the Third People's Hospital of Hainan Province, Sanya, China; 2 Department of Neurosurgery, the Third People's Hospital of Hainan Province, Sanya, China

**Keywords:** Orofacial pain, Trigeminal ganglion, NLRP3 inflammasome, miR-186, Inflammation

## Abstract

The NOD-like receptor family pyrin domain containing 3 (NLRP3) inflammasome is the most frequently studied in the central nervous system and has been linked to neuropathic pain. In this study, a post-translational mechanism of microRNA (miR)-186 via regulating the expression of NLRP3 in the complete Freund's adjuvant (CFA)-treated mice was investigated. The injection of CFA was used to induce trigeminal neuropathic pain in mice. miRs microarray chip assay was performed in trigeminal ganglions (TGs). CFA treatment significantly increased the mRNA expression of NLRP3, interleukin (IL)-1β, and IL-18 in TGs compared to the control group. Moreover, 26 miRs were differentially expressed in TGs from trigeminal neuropathic pain mice, and the expression of miR-186 showed the lowest level of all the miRs. Further examination revealed that NLRP3 was a candidate target gene of miR-186. We delivered miR-186 mimics to CFA-treated mice. The head withdrawal thresholds of the CFA-treated mice were significantly increased by miR-186 mimics injection compared with CFA single treatment. The mRNA and protein expression of NLRP3, IL-1β, and IL-18 in TGs from trigeminal neuropathic pain mice were significantly inhibited by miR-186 mimics treatment compared to the CFA group. miR-186 was able to suppress the neuropathic pain via regulating the NLRP3 inflammasome signaling.

## Introduction

Neuropathic pain is one of the most intractable nervous system diseases and is characterized by spontaneous pain, allodynia, and hyperalgesia (1,2). It has become evident that neuroinflammation is associated with the development of neuropathic pain, which is attributed to inflammatory mediators, including proinflammatory cytokines and chemokines (3,4). Recently, the NOD-like receptor family pyrin domain containing 3 (NLRP3) inflammasome complex (including NOD-like receptor NLRP3, the adaptor molecule apoptosis-associated speck-like protein containing a caspase-recruitment domain, and the effector molecule procaspase-1) play a critical role in the pathogenesis of neuropathic pain (2,5). The NLRP3 inflammasome is the most frequently studied in the central nervous system and has been linked to Parkinson's and Alzheimer's diseases (6,7). Moreover, NLRP3 inflammasome is abundantly expressed in microglia and is closely related to brain functions (8). In the mice spared nerve injury model, the expression of NLRP3 is increased and has a role in acute and chronic pain following peripheral nerve injury (2). Intriguingly, NLRP3 inflammasome is activated in the skin tissue by complete Freund's adjuvant (CFA) injection in rats (9). However, it is unclear whether NLRP3 inflammasome is involved in CFA-induced trigeminal neuropathic pain.

MicroRNAs (miRs) are endogenous noncoding RNAs of 18-25 nucleotides in length and play an important role in controlling gene expression at transcription level or translation level by binding to the target mRNAs 3′-untranslated regions (3′-UTR) (10). Increasing evidence indicates that a large amount of miRs expression in the nervous system is differentially regulated in the development of neuropathic pain (11–14). Specifically, decreased miR-125a-3p contributes to upregulation of p38 MAPK in rat trigeminal ganglions with orofacial inflammatory pain (15). Moreover, the expression of several neuron-specific miRs, including miR-10a, -29a, -98a, and -134a, are decreased in trigeminal ganglions (TGs) of rats with inflammatory muscle pain (16). Interestingly, spinal over-expression of miR-186-5p decreases CXCL13 (a chemokine in microglial activation associated with the pathogenesis of neuropathic pain) expression and alleviates spinal nerve ligation-induced neuropathic pain (1). Notably, the role of miR-186 in trigeminal nerve damage-induced neuropathic pain remains unclear.

In the present study, we used microarray to search for the differential expression of miRs in mice TGs after CFA injection, and we hypothesized that a post-translational mechanism might exist for NLRP3 inflammasome signaling, regulated by miR-186 in a trigeminal nerve damage mice model, to alleviate neuropathic pain.

## Material and Methods

### Cell culture

The ND8/34 cells were purchased from Sigma-Aldrich (USA) and were incubated in Dulbecco's modified Eagle's medium (Thermo Fisher Scientific, Inc., USA) and supplemented with 10% FBS, 100 μg/mL streptomycin, and 100 IU/mL penicillin (all from Sigma-Aldrich) at 37°C in a humidified incubator (Thermo Fisher Scientific, Inc.) with 5% CO_2_, 95% air atmosphere. The medium was replenished every other day.

### Animal treatment

**Figure 1.**
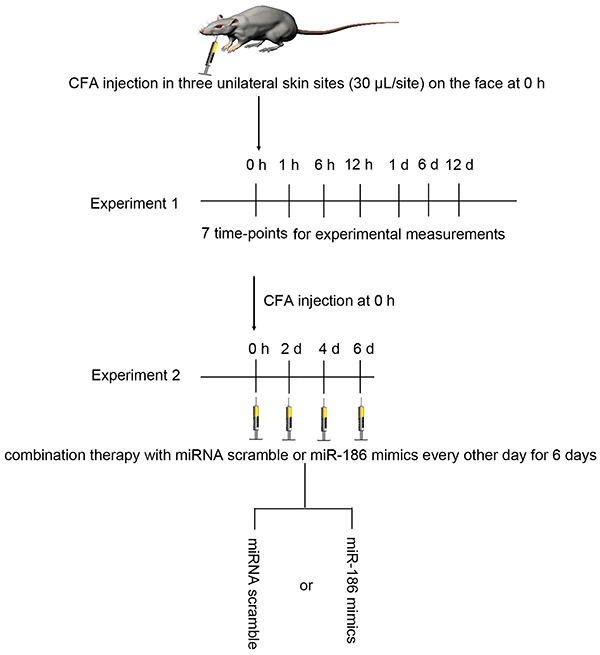
Timeline of the experimental design. CFA: complete Freund's adjuvant.

The experiment was approved by the Ethics Committee of the Third People's Hospital of Hainan Province (China) and was performed in accordance with its guidelines. A total of 96 male C57BL/6J mice (body weight 20±2 g; 8 weeks old) were obtained from the Animal Experimental Center of Hainan Medical College (China) and were allowed to acclimate to the environment for 1 week. The mice were given free access to food and tap water and were caged under controlled temperature (23±2°C) and humidity (55±5%) with an artificial 12-h light/dark cycle. The mice were intraperitoneally injected with pentobarbital sodium (50 mg/kg, Sigma-Aldrich), and then received CFA or normal saline injection. In brief, the CFA (oil:saline, 1:1; Sigma) was subcutaneously injected into three unilateral skin sites (30 μL/site; Figure 1) on the face to induce trigeminal neuropathic pain as previously described (17,18). Mice injected with normal saline were used as controls. In one experiment, the mice received normal saline (control group) or CFA injection (CFA group) at 0, 1, 6, and 12 h, and 1, 6, and 12 days, n=6 in each group at different time-points. In another experiment, the mice received normal saline, CFA, CFA + scramble (60 μg/kg; RiboBio, China) or CFA + miR-186 mimics (60 μg/kg; RiboBio) treatment, n=6 in each group. In the CFA + scramble or CFA + miR-186 mimics treatment groups, the CFA-injected mice were treated with the scramble or miR-186 mimics every other day for 6 days (Figure 1).

### Behavior testing

Head withdrawal threshold is a behavioral parameter and inversely relates to pain. The head withdrawal thresholds were measured by electronic von Frey anesthesiometer (IITC Life Science, USA) at different time-points in mice with normal saline, CFA, or CFA combined with miR-186 mimics injection, as described previously (15).

### Enzyme-linked immunosorbent assay (ELISA)

At various time-points, blood samples (approximately 1.2 mL each mouse) were separated from heart blood and centrifuged at 1200 *g* for 15 min at 4°C. The levels of cytokines, tumor necrosis factor-alpha (TNF-α, Cat. No: E-EL-M0049c), interleukin-1beta (IL-1β, Cat. No: E-EL-M0037c), and interleukin-18 (IL-18, Cat. No: E-EL-M0730c) (Elabscience Biotechnology Co., Ltd, China) were assayed using a double antibody sandwich ELISA following the manufacturer's instructions and evaluated using an ELISA reader (MD SpectraMax M5; Molecular Devices, USA).

### Transfection with miR-186 mimics and inhibitors

The sequences of the miR-186 mimics, inhibitors, and scramble were 5′-GUUUCUUAAGAGGAAAACCCGA-3′, 5′-AGCCCAAAAGGAGAAUUCUUUG-3′ and 5′-CUAAAACCGGCCGUACGGCGUU-3′. The miR-186 mimics, inhibitors, and scramble were synthesized by RiboBio (China). The ND8/34 cells were transfected using lipofectamine 2000 (Invitrogen; Thermo Fisher Scientific, Inc.) at a final concentration of 50 nM. At 24 h post-transfection, the culture medium was replaced and cells were harvested at 48 h for analysis.

### Dual-luciferase reporter gene assay

The potential binding site between miR-186 and NLRP3 was obtained by an online prediction software (miRanda Target database, http://www.microRNA.org), and synthesized by RiboBio. The wild-type (wild) NLRP3-3′-UTR and mutant-type (mut) NLRP3-3′-UTR were inserted into the multiple cloning sites of the luciferase expressing pMIR-REPORT vector (Ambion; Thermo Fisher Scientific, Inc.). For the luciferase assay, the ND8/34 cells (2×10^5^) were seeded into 24-well plates and co-transfected with luciferase reporter vectors containing the wild and mut of NLRP3-3′-UTR (0.5 μg) and miR-186 mimics, inhibitors or scramble (50 nM) using lipofectamine 2000. The luciferase activity was measured using a dual luciferase reporter assay kit (Beyotime Institute of Biotechnology, China), according to the manufacturer's protocol.

### miRs expression profiling

Six mice were randomly divided into normal control group (NC) and CFA group. One day after CFA treatment, TGs from the unilateral surface were removed quickly and immediately frozen in liquid nitrogen for miRs assay. Total RNA in TGs was extracted by TRIzol (Invitrogen; Thermo Fisher Scientific, Inc.). miRs were isolated from total RNA using the miRNA isolation kit (Invitrogen). Denaturing agarose gel electrophoresis was performed using 1% formaldehyde electrophoresis reagent. miRs were labeled with Hy3 or Hy5 fluorescence using the miRCURY™ Array Power Labeling Kit (Exiqon, Denmark) to obtain the fluorescent probe that can be hybridized with the chip. The labeled probe was hybridized with the miRCURY™ chip under the standard condition using the MAUI hybridization system. The fluorescence intensity of the chip was scanned with the Agilent chip scanner and analyzed using Agilent feature extraction software (version 12). The differentially expressed miRs were screened based on the fold change ≥2, P<0.05 and FDR<0.05. Finally, the differentially expressed miRs in TGs were displayed by hierarchical clustering analysis between the two groups.

### Real-time quantitative PCR (RT-qPCR) analysis

Total RNA in TGs was extracted by TRIzol (Invitrogen) according to the manufacturer's protocol. The cDNA was synthesized by reverse transcription reactions with 2 μg of total RNA using moloney murine leukemia virus reverse transcriptase (Invitrogen; Thermo Fisher Scientific, Inc.) according to the manufacturer's protocol. PCR reaction mixtures contained 12.5 μL SYBR Green Supermix (Bio-Rad Laboratory, USA), 1 μL cDNA, 300 nM of each primer, and DEPC H_2_O to a final volume of 25 μL, and then RT-qPCR was performed using the Applied Biosystems 7300 real-time PCR system (Thermo Fisher Scientific, Inc.). The Cq (quantification cycle fluorescence value) was calculated using SDS software, version 2.1 (Applied Biosystems; Thermo Fisher Scientific, Inc.), and the relative expression levels of miRs and mRNA were calculated using the 2^-ΔΔCq^ method (19) and normalized to the internal control U6 and glyceraldehyde 3-phosphate dehydrogenase (GAPDH), respectively. The primers were synthesized by Sangon Biotech (China) and are listed in Table 1.

**Table 1.** miRs and mRNA primers.

	Forward primer (5′-3′)	Reverse primer (5′-3′)
miR-186	CGCGGATCCGGTTTACAGAACACCCATCAT	CCGCTCGAGGTGTTGACATTCACATGCTTC
miR-223	GCGTGTATTTGACAAGCTGAGTT	GTGTCAGTTTGTCAAATACCCCA
miR-721	ACACTCCAGCTGGGCAGUGCAATCAAAAG	TGGTGTCGTGGAGTCG
miR-352	ACACTCCAGCTGGGAGAGTAGTAGGUCGC	TGGTGTCGTGGAGTCG
NLRP3	AAAGCCAAGAATCCACAGTGTAAC	TTGCCTCGCAGGTAAAGGT
IL-1β	TCGCCAGTGAAATGATGGCTTA	GTCCATGGCCACAACAACTGA
IL-18	GACCTTCCAGATCGCTTCCTC	GATGCAATTGTCTTCTACTGGTTC
U6	GCTTCGGCAGCACATATACTAAAAT	CGCTTCACGAATTTGCGTGTCAT
GAPDH	GCACCGTCAAGCTGAGAAC	TGGTGAAGACGCCAGTGGA

### Western blotting

Proteins were extracted from TGs tissues with NP-40 buffer (Beyotime Institute of Biotechnology). Protein concentrations were determined using the Bicinchoninic Acid Kit for Protein Determination (Cat. No: BCA1-1KT; Sigma-Aldrich). Protein (50 μg) for each sample was separated on a 10% SDS-PAGE gel and transferred to nitrocellulose membranes (Bio-Rad Laboratories, Inc., USA). After blocking with 5% non-fat dry milk at room temperature for 2 h, the membranes were incubated with the primary antibody of NLRP3 (Cat. No: ab214185; Abcam), IL-1β (Cat. No: sc-12742; Santa Cruz Biotechnology, USA), and IL-18 (Cat. No: ESAP10527; Elabscience Biotechnology) at room temperature for 2 h. β-actin (Cat. No: sc-130301; Santa Cruz Biotechnology) signals were used to correct for unequal loading. Following three washes with TBST, the membranes were incubated with the appropriate horseradish peroxidase-conjugated secondary antibody (Cat. No: sc-516102; dilution: 1:10,000; Santa Cruz Biotechnology) at room temperature for 2 h and visualized by chemiluminescence (Thermo Fisher Scientific, Inc.). Signals were analyzed with Quantity One¯ software version 4.5 (BioRad Laboratories, Inc., USA).

### Statistical analysis

Data are reported as means±SD for each group. Statistical analyses were performed using PRISM version 5.0 (GraphPad Software, Inc., USA). Inter-group differences were analyzed by one-way analysis of variance, followed by a *post hoc* Tukey test for multiple comparisons. P<0.05 was considered to indicate a statistically significant difference.

## Results

### CFA induced NLRP3 inflammasome signaling activation in trigeminal neuropathic pain mice

**Figure 2.**
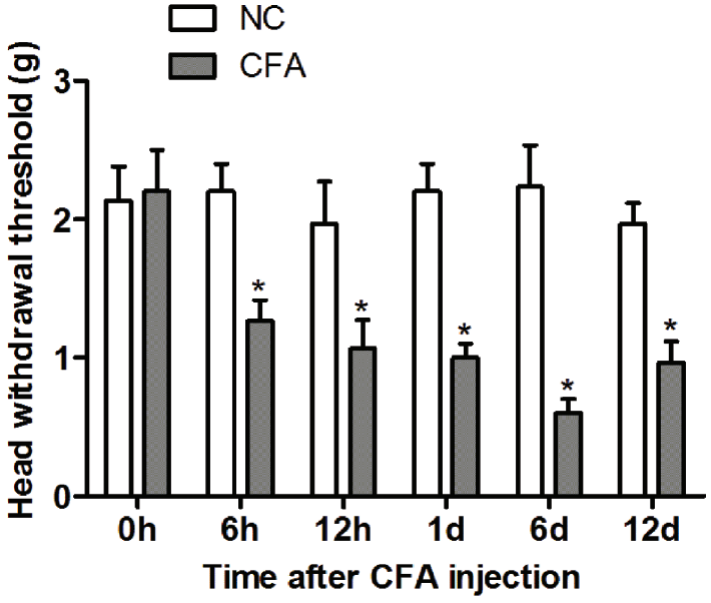
Complete Freund's adjuvant (CFA) induced trigeminal neuropathic pain in mice. The head withdrawal thresholds were measured in ipsilateral orofacial regions at different time-points (n=6). Data are reported as means±SD. *P<0.05 *vs* control group (NC) (ANOVA followed by a *post hoc* Tukey’s test).

**Figure 3.**
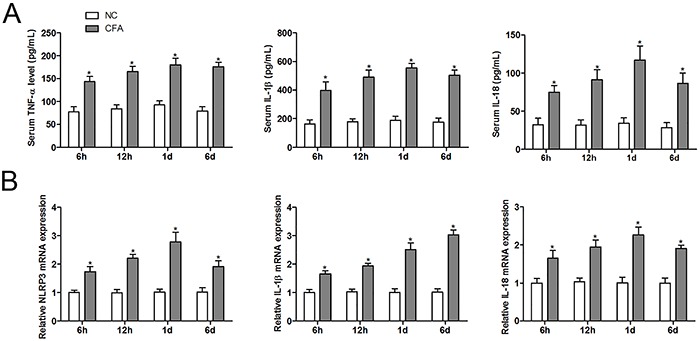
Complete Freund's adjuvant (CFA) activated NLRP3 inflammasome signaling in mice with trigeminal neuropathic pain. Serum levels of tumor necrosis factor-alpha (TNF-α), interleukin-1beta (IL-1β), and interleukin-18 (IL-18) were assayed using a double antibody sandwich ELISA at each time-point (*A*). The mRNA expression of NLRP3, IL-1β, and IL-18 were measured by RT-qPCR at each time-point (*B*) (n=6). Data are reported as means±SD. *P<0.05 *vs* control group (NC) (ANOVA followed by a *post hoc* Tukey’s test).

First, acute mechanical sensitivity was evaluated by Von Frey filament and was comparable between normal control and CFA-treated group at the different time-points (Figure 2). Compared with normal controls, the head withdrawal thresholds of the CFA-treated group were significantly decreased from 6 h (1.27±0.15) to day 12 (0.97±0.12) with the lowest value at day 6 (0.60±0.10). To further determine the association between trigeminal neuropathic pain and neuroinflammation, we measured the levels of proinflammatory cytokines in serum and TGs. Compared with normal controls, the serum levels of TNF-α, IL-1β, and IL-18 in CFA-treated group were significantly up-regulated from 6 h to day 6 with the lowest value at day 1 (Figure 3A). Similarly, CFA treatment also significantly increased the mRNA expression of NLRP3, IL-1β, and IL-18 in TGs compared to the control group (Figure 3B).

### Aberrant miRs expression in TGs from CFA-treated mice

**Figure 4.**
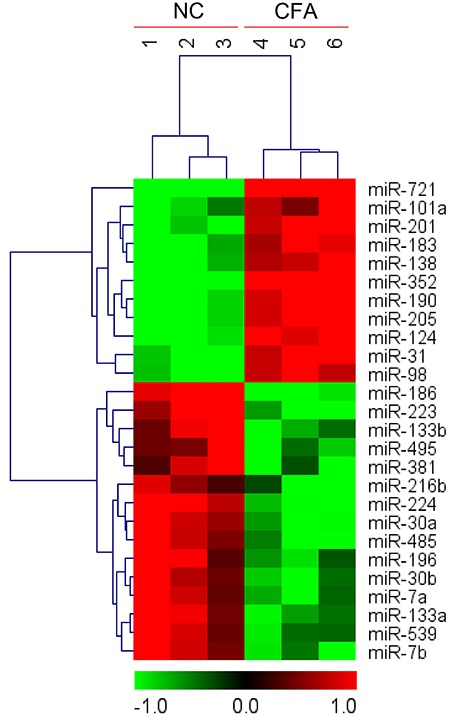
Microarray and hierarchical cluster analysis were performed in trigeminal ganglions (TGs) from complete Freund's adjuvant (CFA)-injected mice and control mice (NC). The figure was drawn by MeV software (version 4.2.6; The Institute for Genomic Research (TIGR), USA). Correlation similarity matrix and average linkage algorithms are used in the cluster analysis. Each row represents an individual miRNA, and each column represents a sample. The color legend at the bottom represents the level of miRNA expression, with red indicating high expression levels and green indicating low expression levels.

**Figure 5.**
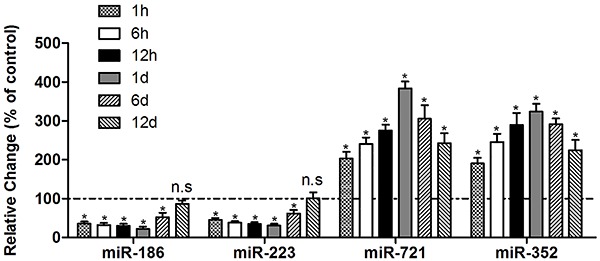
Dysregulated miRs found in trigeminal ganglions from complete Freund's adjuvant (CFA)-injected mice assessed by RT-qPCR at each time-point (n=6). Data are reported as means±SD. *P<0.05 *vs* control group (ANOVA followed by a *post hoc* Tukey’s test). n.s.: not significant.

miRs differential expression was measured by miRNA microarray. Expression profile in the ipsilateral TGs of normal control and CFA-treated mice was detected. The average value for each miR was used for statistics after normalization, and the miR fold change >2 and P value <0.05 was selected. The results demonstrated that 26 miRs were differentially expressed in TGs from trigeminal neuropathic pain mice, among which 11 miRs were increased and 15 were decreased. The expression of miR-186 showed the lowest level of all the miRs, and the expression of miR-721 showed the highest level of all the miRs (Figure 4). Subsequently, the differentially expressed miRs (miR-186, miR-223, miR-721, and miR-352) were further validated by qRT-PCR, and the results indicated that miR-186 and miR-223 were significantly inhibited from 1 h to day 6 in CFA injection mice compared to the control group. Compared to the normal control group, the expressions of miR-721 and miR-352 were up-regulated in all of the experimental points in CFA-treated mice (Figure 5).

### miR-186 targets to NLRP3

**Figure 6.**
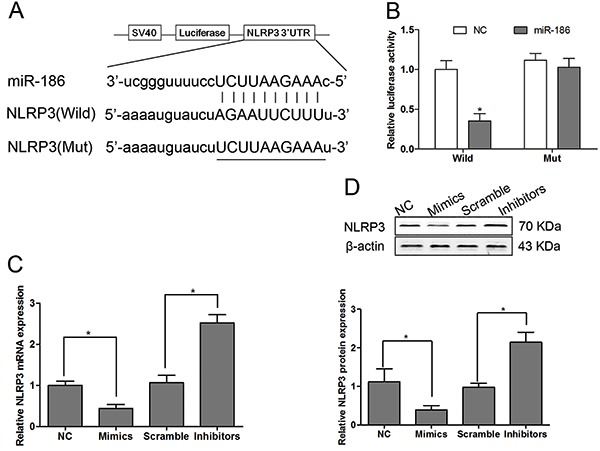
Schematic representation of the putative miR-186 binding site in the 3′UTR of NLRP3 predicted by the miRanda Target database (*A*). The ND8/34 cells were co-transfected with the wild and mutant (Mut) of NLRP3-3′-UTR and miR-186 mimics, inhibitors, or scramble, and the luciferase activity assay was performed after 48 h transfection (*B*). The ND8/34 cells were transfected with miR-186 mimics, inhibitors or scramble, and the mRNA (*C*) and protein (*D*) expressions of NLRP3 were measured by RT-qPCR and western blotting, respectively (n=6 at each time-point). Data are reported as means±SD. *P<0.05 *vs* control group (NC) (ANOVA followed by a *post hoc* Tukey’s test).

Bioinformatics revealed that NLRP3 RNA contained one conserved target site of miR-186, and we found that NLRP3 was a candidate target gene of miR-186. The putative binding sites of murine miR-186 on wild-type NLRP3 3′-UTR was highlighted (Figure 6A). We constructed firefly luciferase reporter vectors containing either wild-type NLRP3 3′UTR or mutation-type NLRP3 3′UTR with modified miR-186 binding site. ND8/34 cells were transfected with either NLRP3 3′UTR wild or mut, along with lentivirus containing miR-186 mimics or its negative control lentivirus (NC). We used a dual-luciferase reporter assay to examine the relative luciferase activities after 24-h transfection. The findings demonstrated that miR-186 mimics significantly reduced the luciferase activity in ND8/34 cells compared with wild-3′UTR control group (P<0.05, Figure 6B). In contrast, with transfection of miR-186 into NLRP3 Mut-type ND8/34 cells, the luciferase activity did not show a significant difference compared with the NC group (Figure 6B). These results suggested that NLRP3 was a direct target of miR-186. The qRT-PCR and western blotting results indicated that miR-186 mimics could significantly suppress NLRP3 mRNA and protein expression. In contrast, down-regulating miR-186 induced the upregulation of NLRP3 mRNA and protein expression in ND8/34 cells (Figure 6C and D).

### Over-expressed miR-186 alleviated trigeminal neuropathic pain

**Figure 7.**
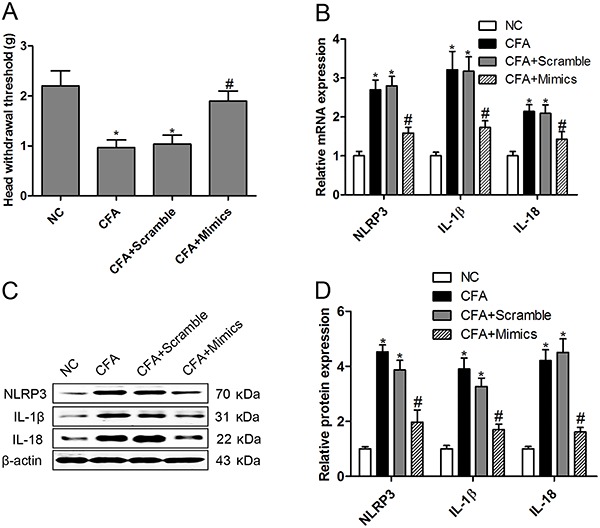
Over-expressed miR-186 alleviated trigeminal neuropathic pain by inhibiting NLRP3 inflammasome signaling. In complete Freund's adjuvant (CFA)-injected mice with miR-186 mimics treatment, the head withdrawal thresholds were measured in ipsilateral orofacial regions (*A*). The mRNA expressions of NLRP3, IL-1β, and IL-18 were measured by RT-qPCR assay (*B*). The protein expressions of NLRP3, IL-1β, and IL-18 were measured by western blotting (*C, D*). Data are reported as means±SD for n=6 in each group. *P<0.05 *vs* control group (NC); ^#^P<0.05 *vs* CFA-injected group (ANOVA followed by a *post hoc* Tukey’s test).

To determine whether inhibition of NLRP3 inflammasome signaling alleviates CFA-induced trigeminal neuropathic pain, we delivered miR-186 mimics to CFA-treated mice. The head withdrawal thresholds of the CFA-treated mice were significantly increased by miR-186 mimics injection compared to CFA treatment alone (Figure 7A). Moreover, the mRNA (Figure 7B) and protein (Figure 7C and D) expression of NLRP3, IL-1β, and IL-18 in TGs from trigeminal neuropathic pain mice were significantly inhibited by miR-186 mimics treatment compared to the CFA group, which indicates that overexpression of miR-186 attenuated trigeminal neuropathic pain in the CFA-induced mice model.

## Discussion

Our study demonstrated that miR-186 was involved in the regulation of trigeminal neuropathic pain and neuroinflammation in a CFA-induced mice model. The levels of miR-186 were significantly decreased in TGs from CFA-treated mice. However, injection of miR-186 mimics significantly attenuated mechanical sensitivity and reduced proinflammatory cytokines (TNF-α, IL-1β, and IL-18) levels. Our study also indicated that miR-186 might alleviate trigeminal neuropathic pain by targeting NLRP3 inflammasome signaling cascade.

A large number of miRs are differentially expressed in a variety of nervous tissues with pain (11,12 [Bibr B13]). Spinal miR-132-3p levels are highest at day 10 after nerve injury model, and increased miR-132-3p expression is associated with chronic neuropathic pain (11). Upregulated miR-195 is found in spinal microglia of rats with spinal nerve ligation (SNL) and it aggravates neuropathic pain by inhibiting autophagy following peripheral nerve injury (20). Moreover, intrathecal miR-96 inhibits Nav1.3 expression and alleviates neuropathic pain in rat following peripheral nerve injury (21). However, there are a few reports of aberrant miRs expression in trigeminal nerve damage-induced neuropathic pain mice. Previous studies show that CFA injection can induce inflammatory muscle pain and inhibit mature miR-10a, -29a, -98, -99a, -124a, -125a-3p, -134, -183, and -299 in TGs (15,16). miRNA microarray analyses show that the levels of miR-155 and miR-223 are significantly increased in the prefrontal cortex of carrageenan-injected mice (22). In our study, the differential expression of miR-186, miR-223, miR-721, and miR-352 was detected in the TGs after orofacial inflammatory pain induced by CFA. However, the expression of some miRs in our study was different from those in previous studies (15,22). Intriguingly, the expression of miR-186 showed the maximal fold change of all the miRs in the TGs after orofacial inflammatory pain induced by CFA. Notably, miR-186-5p is involved in neuropathic pain by regulating CXCL13 mRNA expression following SNL (1). These results suggest that miR-186 plays a crucial role in mice with trigeminal nerve damage-induced neuropathic pain.

Each miR is thought to control hundreds of target genes by targeting mRNAs 3′-UTR (1). Although the known targets of miR-186 have not been completely clarified, research has found that miR-186 targets mitogen-activated protein kinase kinase kinase 2 (MAP3K2) (23), protein phosphatase PPM1B (24), and Jagged1 (25) to regulate cancer cell proliferation and metastasis. Interestingly, miR-186 can target acetylcholine packaging and degradation in neuroinflammation-related disorders (26). Here, we demonstrated a new signaling cascade of miR-186 in mediating trigeminal neuropathic pain by inhibiting NLRP3 inflammasome signaling. In ND8/34 cells, overexpression of miR-186 led to a decreased level of NLRP3 mRNA and protein, and in turn, miR-186 loss-of-function led to a robust up-regulation of NLRP3 mRNA and protein expression. Moreover, overexpression of miR-186 resulted in diminished IL-1β and IL-18 secretion in response to CFA-induced trigeminal neuropathic pain. Previous studies show that NLRP3 inflammasome is involved in neuropathic pain through release of IL-1β (5,27). Immunoexpression of IL-1β is increased in response to chronic constriction injury of the infraorbital nerve in rats (28). A previous study has also found that IL-18 is increased in microglia in the trigeminal spinal subnucleus caudalis after peripheral nerve injury, suggesting a possible role of IL-18 in orofacial neuropathic pain (29).

Taken together, this is the first report that NLRP3 inflammasome signaling as an early molecular response is involved in trigeminal nerve damage-induced neuropathic pain in mice. Our study also indicated that miR-186 was able to suppress the neuropathic pain via regulating the NLRP3 inflammasome signaling.
